# The first complete mitochondrial genome of edible and medicinal fungus *Chroogomphus rutilus* (Gomphidiaceae, Boletales) and insights into its phylogeny

**DOI:** 10.1080/23802359.2021.1950066

**Published:** 2021-07-19

**Authors:** Jia Fu, Wenying Tu, Zhijie Bao, Lijiao Li, Qiang Li

**Affiliations:** a School of Basic Medical Sciences, Chengdu University, Chengdu, P.R. China; b School of Food and Biological Engineering, Chengdu University, Chengdu, P.R. China

**Keywords:** Boletales, mitochondrial genome, phylogenetic analysis

## Abstract

In the present study, we assembled and annotated the complete mitochondrial genome of *Chroogomphus rutilus*. The complete mitochondrial genome of *C. rutilus* was composed of circular DNA molecules, with a size of 37,508 bp. The GC content of the *C. rutilus* mitogenome was 22.82%. A total of 18 protein-coding genes (PCGs), 2 ribosomal RNA (rRNA) genes, and 24 transfer RNA (tRNA) genes were detected in the *C. rutilus* mitogenome. Phylogenetic analysis based on combined mitochondrial gene dataset indicated that the *C. rutilus* exhibited a close relationship with species from the genus *Rhizopogon*. This study served as the first report on the complete mitochondrial genome from the family Gomphidiaceae, which will promote the understanding of phylogeny, evolution, and taxonomy of this important fungal species.

*Chroogomphus rutilus* (Schaeff.) O.K. Mill., 1964, belonging to the order Boletales, is a rare fungal species that grows under pine trees, which could form ectomycorrhiza with plants (Scambler et al. 2018). *Chroogomphus rutilus* is now widely used as a functional food and pharmaceutical product, which has attracted increasing research interests in recent years. Polyphenols extracted from *C. rutilus* exhibited antioxidant, anti-inflammatory and cytotoxic activities (Zhang et al. 2020). Ethanol extract of *C. rutilus* also showed antioxidant, hypoglycemic, hypolipidemic, and antitumor activities (Zhang, Zhao, et al. 2017). Boletales is a highly diverse group. Some species are saprophytic, while others are ectomycorrhizal in the order Boletales (Miller 2003; Wu et al. 2021). It is difficult to classify Boletales species because of its varied and overlapped morphological characteristics (Li, Ren, et al. 2020; Li, Wu, et al. 2021). Mitochondrial genomes have been widely used in the phylogenetic analysis of fungal species (Li, Xiang, et al. 2019; Zhang, Zhang, et al. 2017; Li, He, et al. 2020). However, to date, no complete mitochondrial genome from the family Gomphidiaceae has been reported. In this study, we analyzed the phylogenetic status of *C. rutilus* and the phylogenetic relationships among Boletales species by combining mitochondrial gene set. The complete mitochondrial genome of *C. rutilus* will promote the understanding of phylogeny, evolution, and taxonomy of Boletales species.

The specimen (*C. rutilus*) was collected from Yunnan, China (101.25 E; 25.17 N). A specimen and genomic DNA were deposited at the collection of Yunnan Edible Mushroom Research Initiative of the Yunnan Agricultural University under the voucher number of MG100 (Li et al. 2018). The complete mitochondrial genome of *C. rutilus* was sequenced and *de novo* assembled according to previous described methods (Li, Ren, et al. 2019; Li, Xiang, et al. 2019; Wang, Song, et al. 2020; Wang, Wang, et al. 2020). Briefly, the mitochondrial genome of *C. rutilus* was *de novo* assembled using NOVOPlasty v4.3.1 (Dierckxsens et al. 2017; Li, Ren, et al. 2020). The mitochondrial genome of *C. rutilus* was circularized assembled at the K-mer size of 28. The protein-coding genes, rRNA genes, tRNA genes, and introns of the *C. rutilus* mitochondrial genome were annotated using MITOS (Bernt et al. 2013) and MFannot (Valach et al. 2014), both based on the genetic code 4. We also predicted PCGs or ORFs based on the NCBI Open Reading Frame (ORF) Finder (Coordinators 2017), and annotated by BLASTP searches against the NCBI non-redundant protein sequence database (Bleasby and Wootton 1990). The tRNA genes in the *C. rutilus* mitogenome were also predicted with tRNAscan-SE v1.3.1 (Lowe and Chan 2016).

The complete mitochondrial genome of *C. rutilus* is 37,508 bp in length. The base composition of the *C. rutilus* mitochondrial genome is as follows: A (37.25%), T (39.94%), G (12.07%) and C (10.75%). The complete mitochondrial genome of *C. rutilus* contains 18 protein-coding genes, 2 ribosomal RNA genes (*rns* and *rnl*), and 24 transfer RNA genes (supplemental Fig. S1). No intron was detected in the mitochondrial genome of *C. rutilus* (Zhang and Zhang 2019). To reveal the phylogenetic relationships of Boletales species, we constructed a phylogenetic tree for 20 Boletales species. *Hannaella oryzae* from the order *Tremellales* was set as outgroup (Li, Li, et al. 2021). We used the Bayesian analysis (BI) method to construct the phylogenetic tree for Boletales species based on the combined 14 core protein-coding genes (Cheng et al. 2021; Li, Wu, et al. 2021; Li, Yang, et al. 2020). We first aligned single mitochondrial genes using MAFFT v7.037 (Katoh et al. 2019), and then concatenated these alignments into a gene dataset using the SequenceMatrix v1.7.8 (Vaidya et al. 2011). We detected the best-fit models of evolution and partitioning schemes for the gene dataset using PartitionFinder 2.1.1 (Lanfear et al. 2017). MrBayes v3.2.6 (Ronquist et al. 2012) was used to analyze the phylogenetic relationships of the 20 Boletales species based on the combined gene dataset. As shown in the phylogenetic tree (Figure 1), the mitochondrial genome of *C. rutilus* exhibited a close relationship with species from the genus *Rhizopogon* (Li, Ren, et al. 2019).

**Figure 1.**
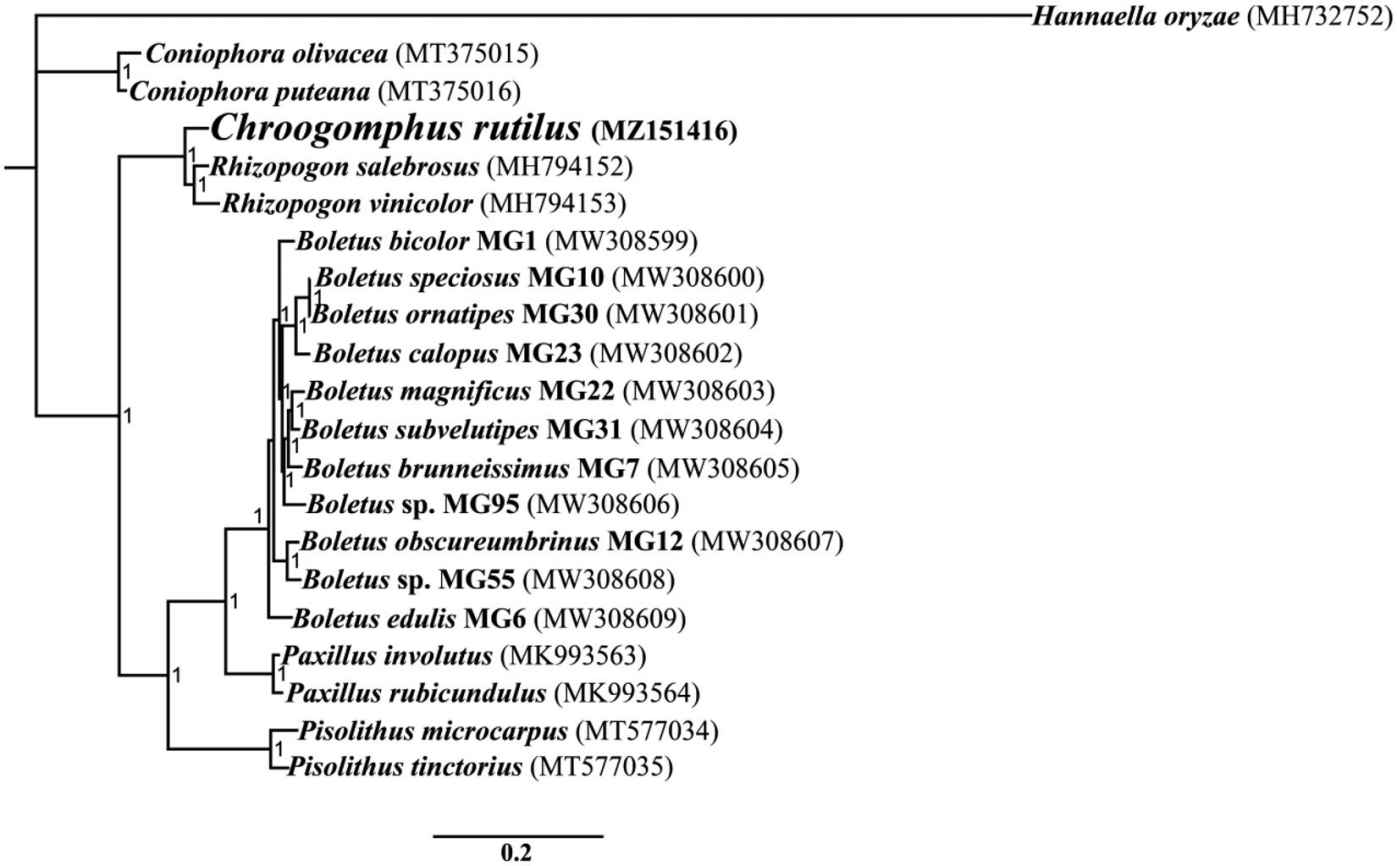
Bayesian phylogenetic analysis of 20 Boletales species based on the combined 14 core protein-coding genes. Accession numbers of mitochondrial sequences used in the phylogenetic analysis are listed in brackets after species.

## Data Availability

The genome sequence data that support the findings of this study are openly available in GenBank of NCBI at (https://www.ncbi.nlm.nih.gov/) under the accession no. MZ151416. The associated BioProject, SRA, and Bio-Sample numbers are PRJNA392574, SRR5804114, and SAMN07303060, respectively.
